# Proteomic analysis in cardiovascular research

**DOI:** 10.1007/s00595-015-1169-4

**Published:** 2015-04-19

**Authors:** Teiji Oda, Ken-ichi Matsumoto

**Affiliations:** Division of Thoracic and Cardiovascular Surgery, Department of Surgery, Shimane University Faculty of Medicine, 89-1 Enya-cho, Izumo, Shimane 693-8501 Japan; Department of Biosignaling and Radioisotope Experiment, Interdisciplinary Center for Science Research, Organization for Research, Shimane University, Izumo, Shimane Japan

**Keywords:** Proteomics, Cardiac valve, Aortic aneurysm, Biomarker, Surgery

## Abstract

Advances in mass spectrometry technology and bioinformatics using clinical human samples have expanded quantitative proteomics in cardiovascular research. There are two major proteomic strategies: namely, “gel-based” or “gel-free” proteomics coupled with either “top-down” or “bottom-up” mass spectrometry. Both are introduced into the proteomic analysis using plasma or serum sample targeting ‘biomarker” searches of aortic aneurysm and tissue samples, such as from the aneurysmal wall, calcific aortic valve, or myocardial tissue, investigating pathophysiological protein interactions and post-translational modifications. We summarize the proteomic studies that analyzed human samples taken during cardiovascular surgery to investigate disease processes, in order to better understand the system-wide changes behind known molecular factors and specific signaling pathways.

## Introduction

Omics-based studies, including genomics, transcriptomics, proteomics, and metabolomics, have been recognized as powerful analytical tools in cardiovascular research. Transcriptomics can analyze mRNA abundance, which cannot be identical to the corresponding protein abundance, as protein abundance is influenced by the balance between synthesis and degradation rates, protein processing, and micro RNA interference [1–5]. This is particularly pertinent to extracellular matrix proteins such as collagen or elastin, which have long half-lives [6]. These proteins support the biological functions of the heart and vessels, including the electrophysiology, contractility, and response to surgical insult. The proteome of diseased tissues such as the aortic aneurysmal wall, calcific aortic valve, or infarcted myocardium can reflect serious changes in protein abundance or protein modifications: namely, post-translational modification [PTM] induced by disease. Many studies have identified potential biomarkers or panels of biomarkers for aortic aneurysms using this technology; however, surgeons who plan to use mass spectrometric measurement, including protein identification and quantification, for their research may find it difficult to understand. In this review, we demonstrate recent scientific evidence identified through cardiovascular proteomics.

## Proteomic strategies

**Fig. 1:**
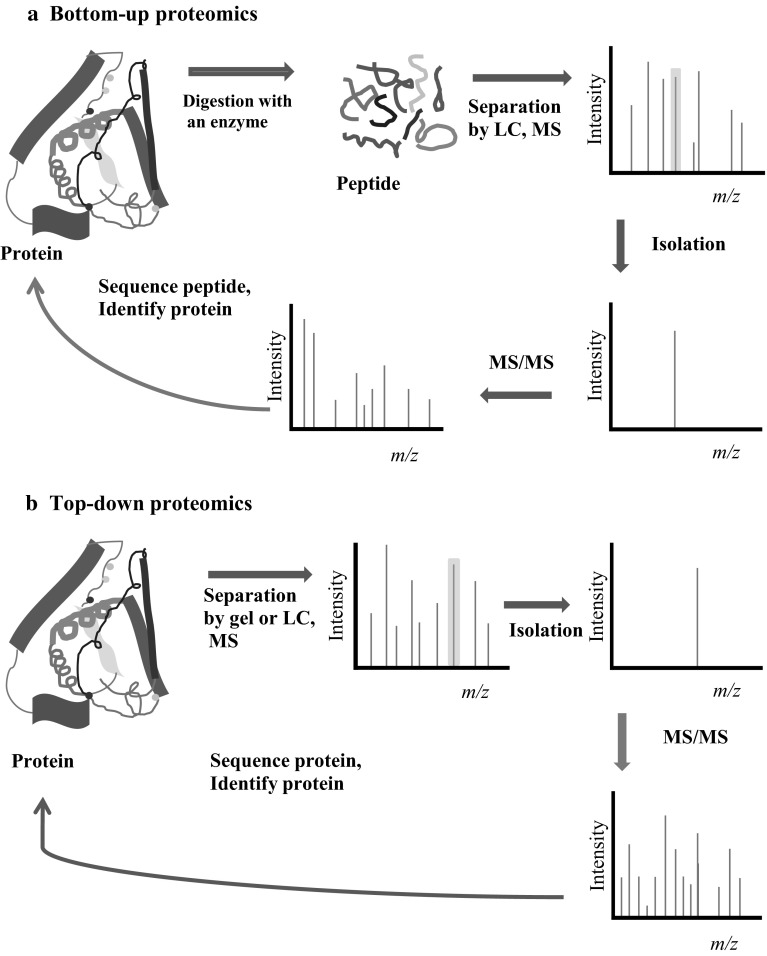
Principal differences between top-down (**a**) and bottom-up (**b**) proteomics. **a** In bottom-up proteomics, protein extracts are separated by 2-dimensional gel electrophoresis (2-DE) and excised gels are digested with chemical tags or separated by liquid chromatography (LC) after protein extracts are digested with chemical tags. Labeled peptides are analyzed and isolated by mass spectrometry (MS) and fragmented by tandem mass spectrometry (MS/MS) to identify the protein from the database. Consequently, hundreds of proteins can be identified and quantified with significant confidences but the sequence coverage of proteins is far from complete sequence coverage. (**b**) In top-down proteomics, a complex sample, such as a tissue sample, is separated by 2-DE or LC and analyzed directly by MS/MS without digestion. Thereby, this strategy can analyze the protein’s full sequence coverage

There are two major proteomic strategies: gel-based proteomics and gel-free proteomics. Both these separation methods are combined with either top-down or bottom-up mass spectrometry (MS) [2–4, 7–9]. In gel-based proteomics, protein extracts are usually separated by 2-dimensional gel electrophoresis (2-DE) or 2-dimensional fluorescence difference gel electrophoresis (2D-DIGE). Selected protein spots are excised and analyzed by tandem mass spectrometry (MS). Gel-based proteomics can visually demonstrate the separated protein spots and quantify protein abundance at the protein level; however, 2-DE has a major limitation in that the gel resolves only proteins larger than 150 kDa within a narrow p*I* range (pH 4–pH 9), indicating a narrow dynamic range of 10^4^ [1, 4, 10]. In gel-free bottom-up analysis, protein extracts are digested into peptides using trypsin and are fractionated by liquid chromatography (LC) before tandem MS, implying that protein abundance is quantified at the peptide level and that a partial sequence of proteins can be recovered and identified [7, 9] (Fig. 1). However, in a newly developed top-down MS, protein is analyzed directly by MS without digestion into peptides and thereby can provide a full sequence protein recovery, which is useful in detecting PTM and isoform composition (so called “proteoform”) [3, 7, 9]. Unlike the well-established bottom-up proteomics, the top-down proteomics is still being developed and necessitates improving protein enrichment and purification, sensitivity and throughput [7, 9, 11]. Currently, bottom-up MS is superior to top-down MS in terms of protein identification and quantification; however, top-down MS is superior to bottom-up MS in terms of protein modification due to complete sequence coverage of the protein [7–9].

The very wide dynamic range of protein abundance is estimated at 10^6^ for cells and tissue, and 10^12^ for plasma [1, 12]. Targeted proteomics has developed progressively to analyze the subcellular fractions or extracellular matrix, aiming to reduce sample complexity and thereby detect low-abundance proteins [11, 13]. This strategy has succeeded in identifying many PTMs using several enrichment methods [14].

## Post-translational modification and cross-talk

In the heart, much of the complexity of protein function arises from PTMs [15]. Van Eyk found that 62 % of 5079 human cardiac proteins studied had at least one PTM, wherein phosphorylation accounts for more than 90 % of all single modification proteins [15]. Acetylation is the next frequently identified PTM, followed by *N*- and *O*-linked glycosylation [15]. These modifications have been reported to occupy the same amino acid residue or adjacent-site residue, and thereby interplay or cross-talk with each other to regulate cardiac function [14–18].

## Abdominal aortic aneurysm and biomarker search using blood sample proteomics

Abdominal aortic aneurysm (AAA) is an asymptomatic disorder, found most commonly in the elderly, which is usually fatal if it ruptures. The risk factors for AAAs include old age, male gender, cigarette smoking, and a family history of aneurysms. Therefore, screening for this catastrophic disease is recommended and has proven to be safe and cost-effective due to high sensitivity and specificity [19, 20]. A systematic review demonstrated that serum elastin peptides and plasmin-antiplasmin complex were strongly associated with AAA expansion and rupture [21]. A recent review and meta-analysis concluded that plasma d-dimer may have a future role as a biomarker [22]. Plasma or serum proteomic studies have demonstrated many other potential biomarkers for the presence of AAA, aneurysm progression, and rupture risk (Table 1). Six proteins (MMP9, CRP, HP, SERPINA1, SERPIN4, PRDX1) have been identified by proteomic studies.

**Table 1:** Biomarker candidates for abdominal aortic aneurysm identified by blood sample proteomics

Study groups	Sample type	Methods	Identified proteins (gene name)	Ref.
AAA vs control	Exosomes and microparticles (plasma)	Label-free quantitative MS	**PLF4**, **FTL**, **CRP**, **OIT3**, **DCD**, **ANXA2**	[64]
ApoE(−/−) mouse	Plasma, aorta	iTRAQ-LC–MS/MS	Eight proteins, including **APOC1**	[65]
Small AAA vs control	Plasma	2D-DIGE MS	**GPI-PLD**, **ITIH4**, *IGHM*, *GSN*, **IGHG1**, **IGHG2**	[66]
AAA vs control	Plasma	SELDI-TOF MS	Serum elastin peptides, plasmin-antiplasmin complexes, MMP9, IFNG, CRP, SERPINA1, lipoprotein (a), IL6	[27]
AAA vs control	Plasma	LC–MS/MS (PAcIFIC MS)	80 proteins, including **ADIPOQ**, **SOD3**, **AMBP**, *SERPIN4*, **CPB2**	[23]
AAA vs control	Serum	2D-DIGE MS	*APOA1*, **GC**, **APCS**, **HP,** *HPX*, **C4A**	[30]
AAA (large/small) vs control	Polymorphonuclear neutrophil, plasma	2D-DIGE MS	41 proteins, including *CAT, TXNRD1*	[24]
AAA (small/large, stable/progressive)	Serum	2D-DIGE MS/MS	**ALB, C3, SERNA1, F12, IGKC**	[25]
AAA vs control	Plasma	2D-DIGE MS	33 proteins, including **fibrinogen, SERPINA1, HP**, *GC, HBB*	[26]
AAA (pre- vs post-operative	Serum	iTRAQ-nanoLC- MS/MS	18 proteins, including **SERPINA4** and **A2** **M**	[67]
AAA vs control	RBC membrane	Label-free quantitative MS	39 proteins, including *CAT* and *PRDX2*	[31]
AAA vs PAD	Macrophage	2D-DIGE, MS/MS with transcriptome	**PRDX1**, *MAPT*, *HSPA8*, *ATP5A1*, *PKM*, *PDIA3*, *GDI2*, *UQCRC2*, *FBP1*, *CAPG*, **GAPDH**, **ACTB**, **CTSS**	[28]
AAA vs control	Serum	2D-DIGE, MS/MS	**PRDX1**	[29]
AAA vs control	Serum	SELDI-TOF–MS	**Hemorphin-7** (HBB)	[68]
AAA AAA vs control	ILT-conditioned medium Serum	LC–MS/MS ELISA	150 proteins, including *CLU, THBS1*	[69]

Upregulated proteins are shown in bold, downregulated proteins are shown in italics, and normal text indicates no available information regarding protein abundance

*AAA* abdominal aortic aneurysm, *ELISA* enzyme-linked immunosorbant assay, *GPI*-*PLD* glycosylphosphatidylinositol-specific phospholipase D, *ILT* intraluminal thrombus, *iTRAQ*-*LC*–*MS/MS* isobaric tags for relative and absolute quantitation-liquid chromatography-mass spectrometry, *PAcIFIC* precursor acquisition independent from ion count, *PAD* Peripheral arterial disease, *RBC* red blood cell, *Ref* references, *2D*-*DIGE* *MS* 2-dimensional fluorescence difference gel electrophoresis, *SELDI*-*TOF* *MS* surface-enhanced laser desorption/ionization mass spectrometry

Two protease inhibitors, α-1-antitrypsin (SERPINA1) and kallistatin (SERPINA4), have been newly identified as potential biomarkers [23–27]. Furthermore, PRDX1, CAT and HP are involved in redox regulation, or are antioxidant proteins, and were detected as possible biomarker candidates from the red blood cell membrane, cultured macrophages, and the serum or plasma of AAA patients [24, 26, 28–31]. However, low-abundance proteins like cytokines are difficult to quantify by conventional untargeted proteomic strategies because of the very wide dynamic range of protein abundance in plasma or serum. At present, immunodepletion of the abundant prions (albumin and immunoglobulin) is commonly adapted to reduce the wide dynamic range of protein abundance [1, 10, 11].

## Pathogenesis of aortic aneurysm and proteomic analysis

Several mechanisms have been reported to be relevant in the pathogenesis of AAA formation: namely, proteolytic degradation caused by the imbalance between several proteases such as matrix metalloproteinases, cathepsins, and serine proteases, and their inhibitors; vascular smooth muscle cell apoptosis and oxidative stress; inflammation and immune responses with leukocyte infiltration modulated by cytokines (IL-1β) or chemokines; biomechanical stress; and genetic components, reported to be present in 20 % of AAA patients [32–34].

Proteomic studies with abdominal aortic wall tissue or intraluminal thrombus (ILT)-conditioned medium have demonstrated many significantly changed proteins (Table 2). These studies have identified PRDX1, PRDX2, thrombospondin (THBS1 or 2), FGA, ACTB, VTN, ANXA2, ANXA5, GAPDH, and COL6A3. Peroxiredoxins (PRDX1, PRDX2) are antioxidant proteins upregulated in ruptured aneurysmal wall tissue and identified by proteomic analysis of intraluminal thrombus in which reactive oxygen species and oxidative stress are enhanced, contributing to aneurysm formation [29, 35, 36]. The C3 and complement pathway are identified by three proteomic studies [29, 36, 37]. Two studies reported a decreased level of C3 in ILT. However, Martinez-Pinna et al. [36] demonstrated increased levels of C3 and proteolytic fragments (C3a/3c/dg), validated by western blot and immunostaining, and found that C3a activates polymorphonuclear cells. Another proteomic study identified increased expression of C4 beta chain in the aneurysmal wall and detected the massive deposition of C1q component by immunohistochemistry [37]. Vitronectin (VTN) is downregulated in the aneurysmal wall. This protein is a cell adhesion and spreading factor and an important member of the integrin family, generally known as an inhibitor of the formation of the membrane attack pathway (the formation of c5b-9 [38]), and is reported to protect matrix proteins against degradation by proteases through binding protease inhibitor PI-1 and clusterin [39]. The annexin family proteins, ANXA1, ANXA2, and ANXA5, are also downregulated in the aneurysmal wall and the inferior mesenteric vein of AAA patients. These calcium-regulated membrane-binding proteins have been reported to have the antithrombotic property of reducing thrombus formation, and thereby regulating the intraluminal thrombus in AAAs [40, 41]. Collagen alpha-3 (VI) chain (COL6A3) was identified in aneurysmal wall tissue in two proteomic studies [39, 42] and downregulated in acute dissecting thoracic aortic samples in a microarray study [43]. An important glycolytic enzyme, glyceraldehyde-3-phosphate dehydrogenase (GAPDH), was downregulated in two proteomic studies and positively correlated with AAA expansion rate in another study [39, 40, 44], indicating failure of aerobic glycolysis to support energy metabolism in the normal aortic wall [40].

**Table 2:** Proteomic analysis of abdominal aortic aneurysmal wall, thrombus, and other tissue samples

Study groups	Sample type	Methods	Identified proteins (gene name)	Ref.
AAA, luminal vs aluminal layer	ILT-conditioned medium	2D-DIGE, MS/MS	**PRDX1** and 31 protein including *complement components*, *thrombospondin*, **FGA**, *HPX*	[29]
AAA, newly formed thrombus vs old thrombus	ILT-conditioned medium	SELDI-TOF–MS	**Hemorphin-7** (in the newly formed luminal thrombus layer compared with the older layer)	[68]
AAA	Aneurysmal wall tissue	2D-DIGE, LC–MS/MS	Nine proteins (including **GAPDH**) associated with AAA expansion rate, three proteins (**GC**, **COL6A3**, *VTN)* associated with AAA size	[39]
Small (3-5 cm) AAA vs large (> 5 cm) AAA	ILT-conditioned medium	Nano LC–MS/MS	257 proteins including *C3* (in large AAA compared with small AAA), coagulation and complement system enriched	[36]
AAA vs control (organ donors)	Aneurysmal wall tissue	2D-DIGE, MS/MS	**SERPINA1**, *ACTC1,ADH1B*, **ALB**, *ANXA2, ANXA5,* **COL6A2**, *CSRP1*, *DSTN*, *ENO1*, **HSP90B1**, **FGG**, *GAPDHS*, *HSPA1A*, **IGHA2**, **IGHG1**, *KRT1*, **CDC40**, *TAGLN*, *TGM3*, **TF**, *VIM*	[44]
AAA, TAA vs non-aneurysmal adjacent aortic tissue	Aneurysmal wall tissue	Nano LC–MS/MS	*Blood coagulation and plasminogen activating cascade in AAA, Integrin signaling pathway in TAA*	[70]
AAA vs control (organ donors)	Aneurysmal wall tissue	2D-DIGE, MS/MS	**Filamin**, *MFAP4*, *ANXA5*, *ANXA2*, *TPI1*, *GAPDH*, *cytosolic aldehyde dehydrogenase*	[40]
AAA, aneurysmal region vs non-aneurysmal region	Aneurysmal wall tissue	2D-DIGE, MS/MS	**C4A**, **ACTB**, **FGB**, **FGA**	[37]
AAA vs control (benign colon disease, left hemi-colectomy)	Inferior mesenteric vein	2D-DIGE, MS/MS	*PHB*, *ANXA1*, *ACTC1*, **VIM**	[71]
AAA vs control (ascending aorta, aortic valve disease, AVR)	ECM proteins of aneurysmal wall tissue and normal thoracic aorta	Nano LC- MS/MS	37 proteins including **collagen XII,** COL6A3, **THBS2**, **AEBP1**, **POSTN**, **FN1**, **TNC**, **MMP12**	[42]
AAA, ruptured vs unruptured	Aneurysmal wall tissue	2D-DIGE, LC–MS/MS	**PRDX2**, **ACTB**, *ALB*, *ACTG2*, *VTN*, *CALR*	[35]

Upregulated genes are shown in bold, downregulated genes are shown in italics, and normal text indicates no available information regarding protein abundance

*AAA* abdominal aortic aneurysm, *ECM* extracellular matrix, *ILT* intraluminal thrombus, TAA thoracic aortic aneurysm, Ref references. *2D*-*DIGE* *MS* 2-dimensional fluorescence difference gel electrophoresis, *SELDI*-*TOF* *MS* surface-enhanced laser desorption/ionization mass spectrometry, *RIPC* remote ischemic preconditioning

Marfan syndrome is caused by a mutation in the fibrillin-1 gene (*FBN*-*1*) and is known to have catastrophic aortic complications including acute aortic dissection and thoracic aortic aneurysm. A comparative proteomic study identified five upregulated proteins expressed in the ascending aorta of Marfan patients, showing upregulation of the C-terminal filamin A and increased activity of calpain by western blotting in the Marfan patients and the bicuspid aortic valve patients [45]. Proteomic analysis using isobaric tags for relative and absolute quantitation (the iTRAQ system) identified lumican as a potential biomarker for acute aortic dissection [46]. Analysis of dissected ascending aortic wall tissues demonstrated the downregulation of alpha-1 antitrypsin and extracellular superoxide dismutase, suggesting that both increased proteolytic damage and oxidative stress play a major role in aortic dissection [47, 48].

## Calcific aortic valve stenosis and proteomic analysis

**Fig. 2:**
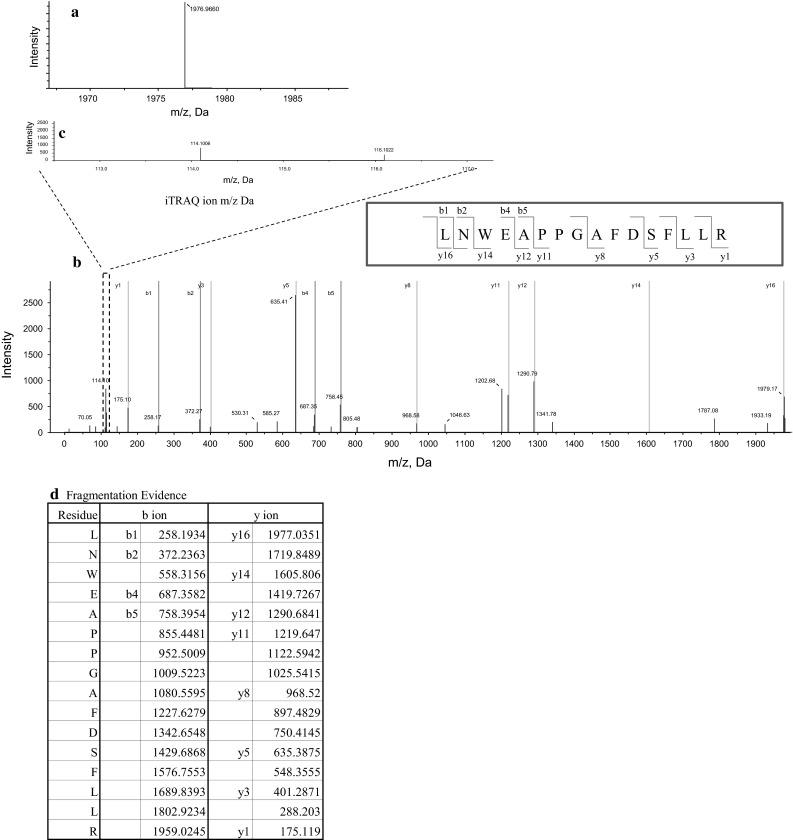
Proteomic analysis of human calcific aortic valve tissue identified tenascin-X protein by nano LC-MALDI-TOF/TOF–MS/MS using Protein Pilot software [54]. The scores of each protein confidence were calculated based on the identified peptide confidences. A representative MS spectrum for the LNWEAPPGAFDSFLLR peptide from tenascin-X protein is shown in **a**. MS/MS spectra: namely, fragmentation spectra are shown in *blue* with matched b-ions (fragment ions extended from the amino terminus) and y-ions (fragment ions extended from the C-terminus) shown in *green* and *red* (**b**), respectively. The quantification evidence is also shown by 114 and 116 iTRAQ reporter ion spectra (**c**) highlighted by the *square with broken lines* in the MS/MS spectra (**b**) and its ratio, demonstrating that protein abundance is measured at the peptide level (bottom-up proteomics). The samples from calcified aortic valve tissues were labeled with a 116 iTRAQ tag, whereas those from adjacent normal aortic valve tissues were labeled with a 114 iTRAQ tag. The iTRAQ ratios were calculated from [116 iTRAQ intensity]/[114 iTRAQ intensity] shown in **c**. The *green* or *red* m/z (Da) figures in **d** show matched ions on the LNWEAPPGAFDSFLLR peptide, which are also shown in **b**

The prevalence of aortic valve stenosis increases by up to 25 % in adults over the age of 65 years [49], and the frequency of surgery for severe calcific aortic valve stenosis also increases with age. Pathological studies of aortic valve stenosis have found dystrophic calcification (83 %), mature lamellar bone with hematopoietic elements (10 %), and active or quiescent osteoblasts (13 %) [50]. Recent studies have demonstrated that the osteogenic transdifferentiation of valve interstitial cells, circulating osteoprogenitors, and the endothelial mesenchymal transition are relevant to the mineralizing cell types causing the pathology of calcific valve disease [51]. Proteomic studies have found that several important proteins, such as gelsolin, are potential biomarkers [52], or biological pathways such as fibrosis, hemostasis, and coagulation [53], as well as blood coagulation and integrin signaling pathways [54] (Table 3). Using the iTRAQ labeling tandem MS, we found that tenascin-X greatly decreased and alpha-2-HS-glycoprotein increased in calcific aortic valves compared with adjacent normal valve tissues (Fig. 2 a–d) [54]. A cluster analysis of 105 identified proteins showed that tenascin-X was linked to the proteins regulating collagen structure and function.

**Table 3:** Proteomic analysis of calcific aortic valves, cardiopulmonary bypass, hypothermia, and remote ischemic preconditioning

Study groups	Sample type	Methods	Identified proteins (gene name)	Ref.
calcific valve tissue vs adjacent normal valve tissue	Aortic valve tissue	Nano LC–MS/MS	**34 proteins including AHSG**, **TTR, APOA1, AGT, FGG,** *39 proteins including TNXB*, *GPX3,HP*	[54]
AS vs control (necropsies)	Aortic valve tissue	2D-DIGE MS/MS	**35 proteins including TTR, APOA1, FGG,** *8 proteins including GPX3,HP*	[53]
AS vs control (autopsies)	Cultured medium from aortic valve tissue, plasma	Nano LC–MS/MS	50 proteins including **AGT,GSN,** *TNXB* in cultured medium, **GSN** in plasma	[52]
Post vs pre CPB	Plasma	2D-DIGE-LC–MS/MS	*HP, CLU, TTR,* **SERPINA3, LRG1, APOE**	[55]
Post vs pre pediatric CPB	Plasma	2D-DIGE MS/MS	HPX, **SERPINA3**, *A2* *M, ITIH4, C3, APOA4, APOE, APOA1,CP*	[75]
Post vs Pre CPB, AKI vs non-AKI	Urine	SELDI-TOF–MS	**B2** **M,** *HAMP (hepcidin*-*25)* in AKI patients compared with non-AKI	[76]
Post and pre CPB, AKI vs non-AKI	Urine	2D-DIGE MS/MS	**AZGP1,LRG1, MASP2, HSPG2, Ig kappa chain, RBP4, AMBP,** *UMOD* in post CPB, *AZGP1, AMBP* in AKI	[77]
Piglet CPB with DHCA vs sham	Cerebral neocortex, plasma	2D-DIGE MS/MS	*3 proteins including APOA1,* **3 proteins** in cerebral tissue, **APOA1** in plasma	[56]
CPB with DHCA vs normothemic CPB	Plasma	nano LC–MS/MS	**Complement activation, proteolysis** in normothermic CPB, **Complement activation, proteolysis** after rewarming in DHCA	[57]
After vs before RIPC, human	Plasma, taken form ischemic arm	2D-DIGE MS/MS, LC–MS/MS	48 up or down-regulated proteins including acute phase response and immune response	[62]
RIPC vs sham, mice	Ventricular tissue	LC–MS/MS, with phospho-peptide enrichment	**Phosphoproteins in the Z-disk including phosphomyozenin-2 (Myoz2)**	[63]
RIPC vs sham, rat	Plasma, taken from IVC	SELDI-TOF–MS	**AOPA1**	[78]

Upregulated genes are shown in bold, downregulated genes are shown in italics, and normal text indicates no available information regarding protein abundance

*Ref* references, *LC*–*MS/MS* liquid chromatography-mass spectrometry, *AS* aortic stenosis, *2D*-*DIGE* *MS* 2-dimensional fluorescence difference gel electrophoresis, *CPB* cardiopulmonary bypass, *AKI* acute kidney injury, *SELDI*-*TOF* *MS* surface-enhanced laser desorption/ionization mass spectrometry, *DHCA* deep hypothermic circulatory arrest, *IVC* Inferior vena cava, *RIPC* remote ischemic preconditioning

## Cardiopulmonary bypass, hypothermia, and remote ischemic preconditioning

**Fig. 3:**
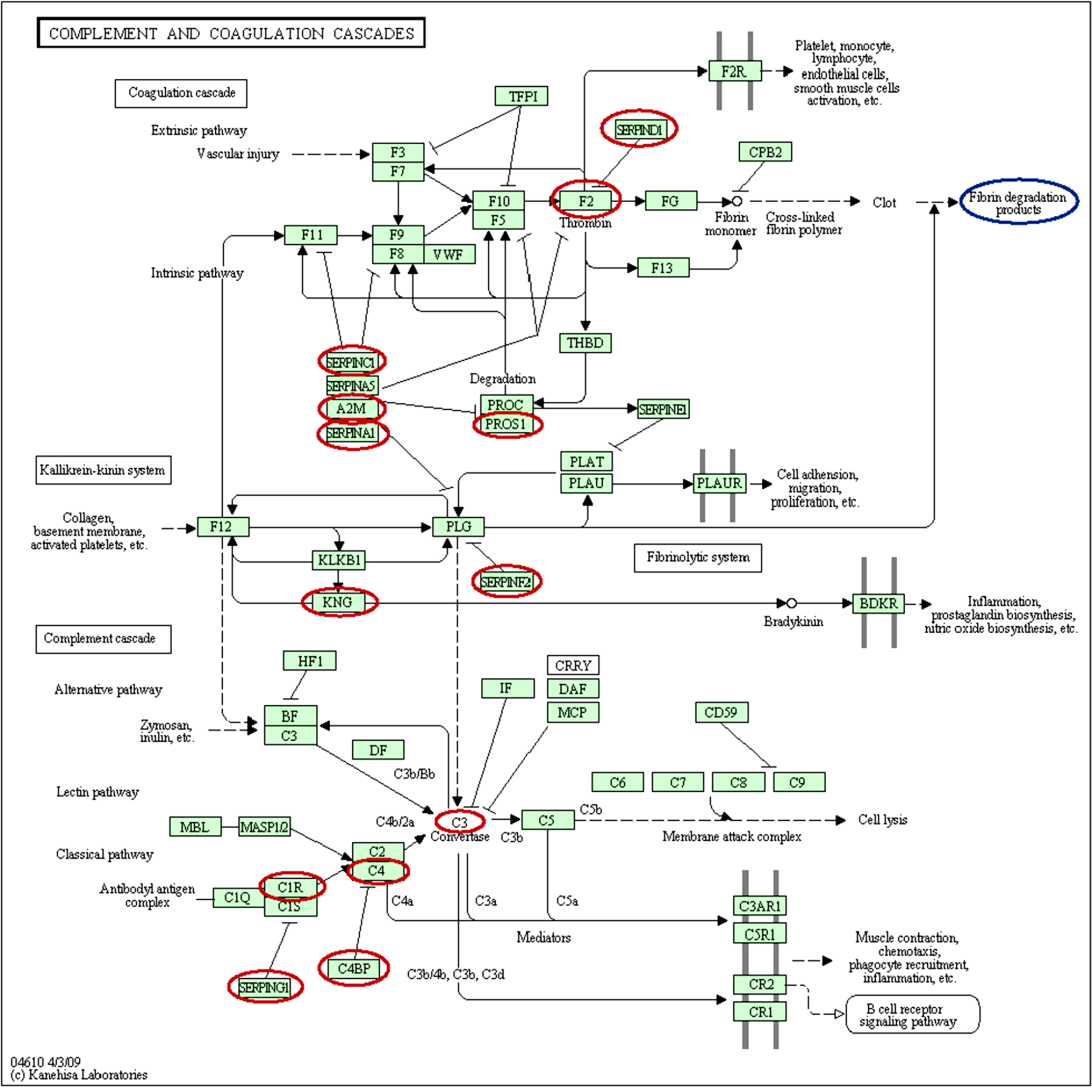
Coagulation cascades, the kallikrein–kinin system, and complement cascades interact with each other. By analyzing plasma from patients undergoing aortic surgery during hypothermic and normothermic cardiopulmonary bypass (CPB), proteomics revealed 13 proteins (*red circles* on the pathway map) on the Kyoto Encyclopedia of Genes and Genomes (KEGG, http://www.kegg.jp/kegg) [57]. The standard clinical tests for biocompatibility of CPB are FDP and d-dimer (*blue circles* on the map), indicating that these tests measure the final products of these cascades, but that proteomic analysis can quantitatively detect protein expressed differently during the interaction process

Cardiopulmonary bypass (CPB) and hypothermia have been utilized in cardiovascular surgery for more than 50 years, but their profound and pleiotropic effects remain to be fully elucidated. The proteomic approach has been receiving much attention in this clinical area. Proteomic analyses of plasma taken from patients undergoing coronary artery bypass grafting with CPB revealed that a protease/antiprotease imbalance develops after surgery, with early activation of cathepsin G (a serpin involved both in inflammation and coagulation activation), and then a delayed increase in alpha 1-antichymotrypsin (an inhibitor of neutrophil cathepsin G) [55] (Table 3). This imbalance is consistent with the postoperative systemic inflammatory response and dysregulation of hemostatic balance. Although deep hypothermic circulatory arrest is used in complex congenital or aortic arch surgery aiming for cerebral protection during circulatory arrest, the mechanism of protection of hypothermia against cerebral ischemia is not fully understood. Proteomics of the cerebral cortex and plasma newly identified six proteins expressed differently in an animal model. Sheikh et al. [56] concluded that the plasma apolioprotein A-1 level may be a new potential biomarker of cerebral injury. Exposure of blood components to the CPB circuit activates blood cells, endothelial cells, and proteins, resulting in the dysregulation of multiple organs and leading to postoperative complications. We investigated this biological response by comparative proteomic analysis between normothermic and deep (22 °C) hypothermic CPB in aortic surgery [57]. The CPB-induced complement activation was suppressed by deep hypothermic CPB compared with normothermic CPB, suggesting that deep hypothermia could improve the biocompatibility of the CPB circuit. The complement cascade has been reported to interact with both the coagulation cascade and the kallikrein–kinin system [58]. We identified 13 proteins belonging to the complement and coagulation cascades, with abundances as demonstrated in the pathway map of the Kyoto Encyclopedia of Genes and Genomes (KEGG, http://www.kegg.jp/kegg) (Fig. 3). These data are thought to be important in comprehensively evaluating the biocompatibility of the CPB circuit, as previously evaluated by the levels of the final product, such as fibrin degradation products.

Brief episodes of distal organ ischemia can protect the heart against ischemia. This phenomenon is called remote ischemic preconditioning (RIPC) [59] and it has been successfully translated into coronary artery bypass surgery, where RIPC was proven as an effective method in perioperative cardiac protection and improved patient prognosis [60]. The effects of RIPC could be produced via systemic release of an unknown cardioprotective factor [61]. Plasma proteomics using both 2D-DIGE MS and liquid chromatography–mass spectrometry identified 6 and 48 proteins, respectively, which were differentially regulated in blood taken from the ischemic arm, but did not identify the protein that provided cardioprotection [62] (Table 3). Cardiac phosphoproteomics revealed upregulation of the phosphorylation of Z-disk proteins, including phosho-myozenin-2, during RIPC in an animal study [63]. These studies indicate that proteomics could help to explore the underlying mechanism through unbiased searches at the protein level, obtaining a “system-wide perspective”. However, this is not enough to enable us to detect a unique target protein because of the wide dynamic range of protein abundance, requiring further technology in mass spectrometry. Future targeted proteomics using multiple-reaction monitoring MS (MRM-MS) could help us overcome this obstacle [6]. Multiple-reaction monitoring, also known as selected reaction monitoring (SRM), is generally performed with the triple quadrupole instrument. The specific m/z selection of precursor ions from the target protein is done in the first quadrupole, the analytes are fragmented in the second quadrupole, and the product ions are filtered through the m/z selection in the third quadrupole, leaving only a particular fragment for specific detection. This process results in higher sensitivity, better quantitative accuracy, and wider dynamic range in target proteomics [11, 72–74]. Top-down proteomics can also be employed as a targeted proteomic technique in cardiovascular research [9]. However, the top-down proteomics is still a developing method designed to improve separation of intact proteins, sample preparation, sensitivity/detection limits, and the detection of large proteins (>60 kDa) [7–9].

## Conclusion

This review highlights proteomic analysis in cardiovascular research, analyzing the sample taken during cardiovascular surgery. Blood samples, aneurysmal wall tissue, calcific valve tissue and myocardial tissue are effectively utilized by proteomics to quantify hundreds of protein expressions and changes in post-translational modification that could lead to deteriorated cardiac function or cardiovascular diseases. Despite rapidly developing mass spectrometry technology and internet-based bioinformatics tools, investigation of the wide dynamic range of protein abundance and PTMs presents many challenges. Researchers should select methodologies such as gel-based or gel-free, top-down or bottom-up proteomics most appropriate for their study designs.
